# Heterozygous Disruption of *Autism susceptibility candidate 2* Causes Impaired Emotional Control and Cognitive Memory

**DOI:** 10.1371/journal.pone.0145979

**Published:** 2015-12-30

**Authors:** Kei Hori, Taku Nagai, Wei Shan, Asami Sakamoto, Manabu Abe, Maya Yamazaki, Kenji Sakimura, Kiyofumi Yamada, Mikio Hoshino

**Affiliations:** 1 Department of Biochemistry and Cellular Biology, National Institute of Neuroscience, NCNP, Tokyo, Japan; 2 Department of Neuropsychopharmacology and Hospital Pharmacy, Nagoya University Graduate School of Medicine, Nagoya, Japan; 3 Department of Cellular Neurobiology, Brain Research Institute, Niigata University, Niigata, Japan; Rikagaku Kenkyūsho Brain Science Institute, JAPAN

## Abstract

Mutations in the *Autism susceptibility candidate 2 gene* (*AUTS2*) have been associated with a broad range of psychiatric illnesses including autism spectrum disorders, intellectual disability and schizophrenia. We previously demonstrated that the cytoplasmic AUTS2 acts as an upstream factor for the Rho family small GTPase Rac1 and Cdc42 that regulate the cytoskeletal rearrangements in neural cells. Moreover, genetic ablation of the *Auts2* gene in mice has resulted in defects in neuronal migration and neuritogenesis in the developing cerebral cortex caused by inactivation of Rac1-signaling pathway, suggesting that AUTS2 is required for neural development. In this study, we conducted a battery of behavioral analyses on *Auts2* heterozygous mutant mice to examine the involvement of *Auts2* in adult cognitive brain functions. *Auts2*-deficient mice displayed a decrease in exploratory behavior as well as lower anxiety-like behaviors in the absence of any motor dysfunction. Furthermore, the capability for novel object recognition and cued associative memory were impaired in *Auts2* mutant mice. Social behavior and sensory motor gating functions were, however, normal in the mutant mice as assessed by the three-chamber test and prepulse inhibition test, respectively. Together, our findings indicate that AUTS2 is critical for the acquisition of neurocognitive function.

## Introduction

Acquisition of the higher cognitive functions in mammalian brain is primarily accomplished by a sequence of neurodevelopmental events comprised of cell differentiation and migration followed by cell morphogenesis. The cellular changes include extension and branching of neurites and formation of synapses to assemble the proper neural circuits during brain development. Genetic defects involving these developmental processes lead to a variety of neuropsychiatric illness such as autism spectrum disorders (ASDs), intellectual disability (ID), schizophrenia and epilepsy.

Disruption of the *Autism susceptibility candidate 2* gene (*AUTS2*) by de novo balanced translocation, was originally associated with ASDs, but more studies have shown that it is also associated with a wide range of neurological diseases such as ID, attention deficit hyperactivity disorder (ADHD), dyslexia and epilepsy [1–7]. Moreover, it has recently been reported that *AUTS2* is also associated with schizophrenia as well as drug dependence including alcohol and heroin [8–11]. Therefore, this gene may be involved with multiple psychiatric disorders rather than only associated with a particular type of ASD [2].

*Auts2* is highly expressed in several brain regions such as the cerebral cortex, hippocampus and cerebellum [12]. In particular, *Auts2* is enriched in the prefrontal cortex compared with the caudal part of cerebral cortex in adult mice brain, implying the involvement of AUTS2 for higher order brain function in mammals [12–14].

AUTS2 encodes a protein that lacks characteristic functional protein domains or protein-binding consensus motifs with the exception of two proline-rich regions (PR1 and PR2) and several putative nuclear localization sequences [2, 12]. Although the precise function of AUTS2 has long remained unclear, the knockdown of Auts2 in zebrafish by morpholino exhibited microcephaly with a decrease of neurons in the developing brain, suggesting that AUTS2 is required for proper neural development [2]. Furthermore, Gao *et al* demonstrated that nuclear AUTS2 participates in the transcriptional control of multiple genes for neural development by interacting with the Polycomb group protein complex [15]. Recently, we have also demonstrated that AUTS2 is not only localized at cell nuclei but also cytoplasm, and that cytoplasmic AUT2 plays an important role in regulating cytoskeletal dynamics, acting as the upstream factor for the Rho family small GTPase Rac1 and Cdc42 in neurons in the developing cerebral cortex [16]. AUTS2 activates the Rac1-signaling pathway via the PR1 domain to induce lamellipodia in neuroblastoma cells as well as promoting neurite extensions of hippocampal primary cultured neurons [16]. In contrast, AUTS2 acts as a suppressor against Cdc42 to inhibit filopodia formation. Furthermore, we found that the AUTS2-Rac1 signaling pathway is required for neuronal migration and subsequent neuritogenesis in the developing cerebral cortex, indicating that AUTS2 plays a key role in brain development [16].

In this study, we performed a battery of behavioral tests to examine whether disruption of the *Auts2* gene affects physiological brain functions. We found that *Auts2* heterozygotic mutant mice displayed behavioral abnormalities in anxiety-related emotions and memory functions, indicating the role of AUTS2 in the acquisition of neurocognitive function as well as cortical development.

## Materials and Methods

### Experimental Animals

Generation of *Auts2* ^*Neo*^ mice and genotyping for the *Auts2*-floxed allele have been previously described [16]. Mice for all behavioral tests were generated by crossing *Auts2* heterozygous male mice with wild type C57BL6/N female mice (Charles River Laboratories, Japan) to exclude the possibility that altered behaviors in the mutant dams could influence the postnatal development of their offspring as well as their future behavior. Pups were kept with their dams until weaning at postnatal day 21. After weaning, male mice were cohoused in same-genotype groups of 2–4 littermates per cages before and during the behavioral tests. Animals at 3 months of age were transferred for behavioral testing. Mice were maintained in ventilated racks with a 12 hr light/dark schedule with food and water *ad libitum* in temperature controlled, pathogen-free facilities. All animal experiments in this study have been approved by the Animal Care and Use Committee of the National Institute of Neuroscience, Japan, and the guidelines established by the Institutional Animal Care and Use Committee of Nagoya University, the Guiding Principles for the Care and Use of Laboratory Animals approved by the Japanese Pharmacological Society, and the National Institutes of Health Guide for the Care and Use of Laboratory Animals. All efforts were made to minimize suffering and to reduce the number of animals used.

### Behavioral analysis

For all behavioral analyses, *Auts2* ^*Neo/+*^ heterozygotes and their wild type littermate male mice (5–8 months old) were used. Behavioral analyses were carried out using the same set of mice (Auts2^Neo/+^ = 14, WT = 17 mice) in the following sequence: locomotor activity, open field test, novel object recognition test, elevated-plus maze test, social interaction test, prepulse inhibition test, and fear conditioning test. For the rotarod test, in which separate cohort of mice was used (Auts2^Neo/+^ = 9, WT = 10 mice).

### Locomotor activity

Locomotor activity tests were carried out as described previously [17]. We used a black frosted plexiglass-bottom transparent acrylic box (25 cm length, 25 cm wide, 20 cm height) equipped with a digital counter with an infrared sensor (BrainScience Idea, Osaka, Japan). The light condition was set at 15 lx in an experimental room. Locomotor activity was monitored every 5 min for 60 min.

### Open field test

Open field tests were carried out as described previously [18]. The size of the apparatus was 60 cm diameter and 35 cm height. The floor was divided into an inner circle with a diameter of 40 cm and an outer sector encompassing the inner area. The light condition was set at 60 lx in an experimental room. Mice were placed in the center of an open field, and their movement of mice was monitored via a camera mounted above the apparatus for 5 min. The activity including distance and time spent in each sector was analyzed by the ethovision automated tracking program (Brainscience Idea Co. Ltd., Osaka, Japan).

### Elevated-plus maze test

The elevated-plus maze consisted two open and two closed arms emanating from a common central platform and was located to 50 cm above floor level [19]. The size of each component was as follows: open arm, 25 cm length, 8 cm wide, 0.5 cm height; closed arm, 25 cm length, 8 cm wide, 20 cm height; central platform 8 cm length, 8 cm wide. The light condition was set at 170 lx in an experimental room. Mice were placed on the central platform of the maze facing an open arm, and the duration of time spent in an arm and number of arm entries was monitored for 5 min. An arm entry was defined when all four paws entered the arm.

### Novel object recognition test

A novel object recognition test was carried out as described previously [20]. The apparatus consisted of an acrylic box and objects. The size of the acrylic box was 30 cm length, 30 cm wide and 35 cm height. The objects used in this study were a golf ball, square pyramid, and wooden cylinder, which were different in shape and color but similar in size. The light condition was set at 16 lx in an experimental room. The habituation session was carried out for 3 days. Each mouse was put in an open box and allowed to explore for 10 min. For the training session, two novel objects were placed in the open box and the exploring activity of the mouse was monitored for 10 min by using video camera. The time spent exploring each object was analyzed in a double-blind manner. The exploring behavior was defined when the mouse was facing, touching or sniffing the object. The retention session was carried out 24 h after the training session. For this, two objects were located in the open box, but a novel object replaced one of the familiar objects used during training. The exploring activity was monitored for 5 min. To measure cognitive function, we calculated the percentage of the amount of time spent exploring the novel object to the total time spent exploring both objects as exploratory index.

### Fear conditioning test

The fear-conditioning test was carried out as described previously [21]. In the conditioning phase, we used a metal floored conditioning chamber equipped with a tone generator. The size of the chamber was 30 cm length, 30 cm wide and 40 cm height (Brainscience Idea Co. Ltd.). A 15-sec white noise tone at 85 dB (conditioned stimulus) was delivered, and then an electrical foot shock of 0.8 mA (unconditioned stimulus) was delivered during the last 5 sec of the conditioned stimulus. The conditioning was repeated four times at 15-sec intervals and the freezing response was measured during each interval. The context-dependent test was carried out 24 hr after the conditioning. The freezing response was measured for 2 min in the absence of the conditioned stimulus. Tone-dependent test was carried out 4 hr after the context-dependent test. During the tone-dependent test, mice were put in a standard transparent rectangular rodent cage and delivered a continuous-tone stimulus identical to the conditioned stimulus. The freezing response was measured for 1 min.

### Social interaction test

Social interaction tests were carried out as described previously [22]. The apparatus used in the study was a black plexiglass rectangular box and two identical clear Plexiglas cylinders. The size of box was 52 cm length, 25 cm wide and 23 cm height. There were three interconnected chambers in the box. The size of both end chambers was 19 cm length by 25 cm wide. The size of center the chamber was 12 cm length by 25 cm wide. The size of the cylinder was 7 cm in diameter, 12 cm height, and the side of cylinder had multiple 1-cm holes in diameter, evenly spaced over the entire surface. The light condition was set at 20 lx in an experimental room. During habituation, empty cylinders were placed in each end chamber. A mouse was put in the center chamber and its behavioral approach to the chambers was monitored for 10 min. During the sociability test, an unfamiliar male C57BL/6 mouse (stranger 1) that had no prior contact with the test mouse was placed in one of the empty chambers, and the behavioral approach to the empty chamber and stranger 1 was monitored for 10 min. During the social novelty test, new unfamiliar male C57BL/6 mouse (stranger 2) was placed in another chamber, and the behavioral approach to the stranger 1 and stranger 2 was monitored for 10 min. The amount of time spent in each zone was measured by an ethovision automated tracking program (Noldus. Wageningen, Netherlands). A zone was defined as area surrounding the plexiglass cylinder (19 cm in diameter). Data from animals that did not approach any chamber were excluded.

### Prepulse inhibition (PPI) test

The PPI test was carried out as described previously [23]. Mice were habituated to the chamber (San Diego Instruments, San Diego, California) with 65 dB background noise. After habituation, mice were subjected to 10 startle trials followed by 10 no-stimulus trials, and then 40 PPI trials were started. The intertrial interval was pseudo-randomly selected 10–20 sec and the total session was completed within 17 min. In the startle trial, white noise at 120 dB was presented for 40 msec. During PPI trials, a prepulse (20 msec white noise at 69, 73, 77 or 81 dB) was inserted 100 msec before the startle stimulus (120 dB, 40 msec white noise). Each of the four prepulse trials (69, 73, 77 or 81 dB) was repeated 10 times. The resulting movement of the mouse in the startle chamber was monitored for 100 msec after startle stimulus onset (sampling frequency 1 kHz) and the maximal response was analyzed. Basal startle amplitude was defined as the mean amplitude of the 10 startle trials. PPI (%) was calculated according to the following formula: [1−(mean amplitude of the 10 PPI trials / basal startle amplitude)] ×100. Data from animals that did not demonstrate startle response were excluded.

### Rotarod test

Mice were placed on an accelerating rotarod (Rotarod 370, Bio Media) from 6 to 42 rpm for 30 sec and the time was measured until they fell from a 3.0 cm-diameter rotating drum. If the mouse was able to remain on the rod over 30 sec, the trial was terminated. The mice in each group were subjected to one trial a day for five consecutive days.

### Statistical analysis

Two-way analysis of variance (ANOVA) with repeated measurements followed by the Bonferroni test was used for multiple-group comparisons (locomotor activity, rotarod test, novel object recognition test and prepulse inhibition test). Two-way analysis of variance (ANOVA) followed by the Bonferroni test was used for multiple-group comparisons (social interaction test). Wilcoxon matched-pairs signed rank test was used for paired two-group comparisons. Mann-Whitney U-test was used for non-paired two-group comparisons.

## Results

We performed a battery of behavioral tests using *Auts2* ^*neo/+*^ heterozygous mice, which express only ~50% levels of AUTS2 protein compared to wild type and exhibit mild abnormalities in neuronal migration and neurite extension [16]. Because in most human patient cases, abnormalities of the *AUTS2* locus are heterozygous not homozygous, we predicted that *Auts2* ^*neo/+*^ heterozygotes might represent a good animal model for *AUTS2*-related neuropsychiatric disorders. Overall, *Auts2* mutant mice appeared grossly healthy and exhibited no obvious differences in physical characteristics, with the exception of a slight decrease in body weight compared with WT littermates. (The body weight measured at 8 month of age; Auts2^Neo/+^, 39.08 ± 1.02 g (n = 13); WT, 34.46 ± 0.83 g (n = 13); p<0.01; data are mean ± SEM, t-test).

**Fig 1:**
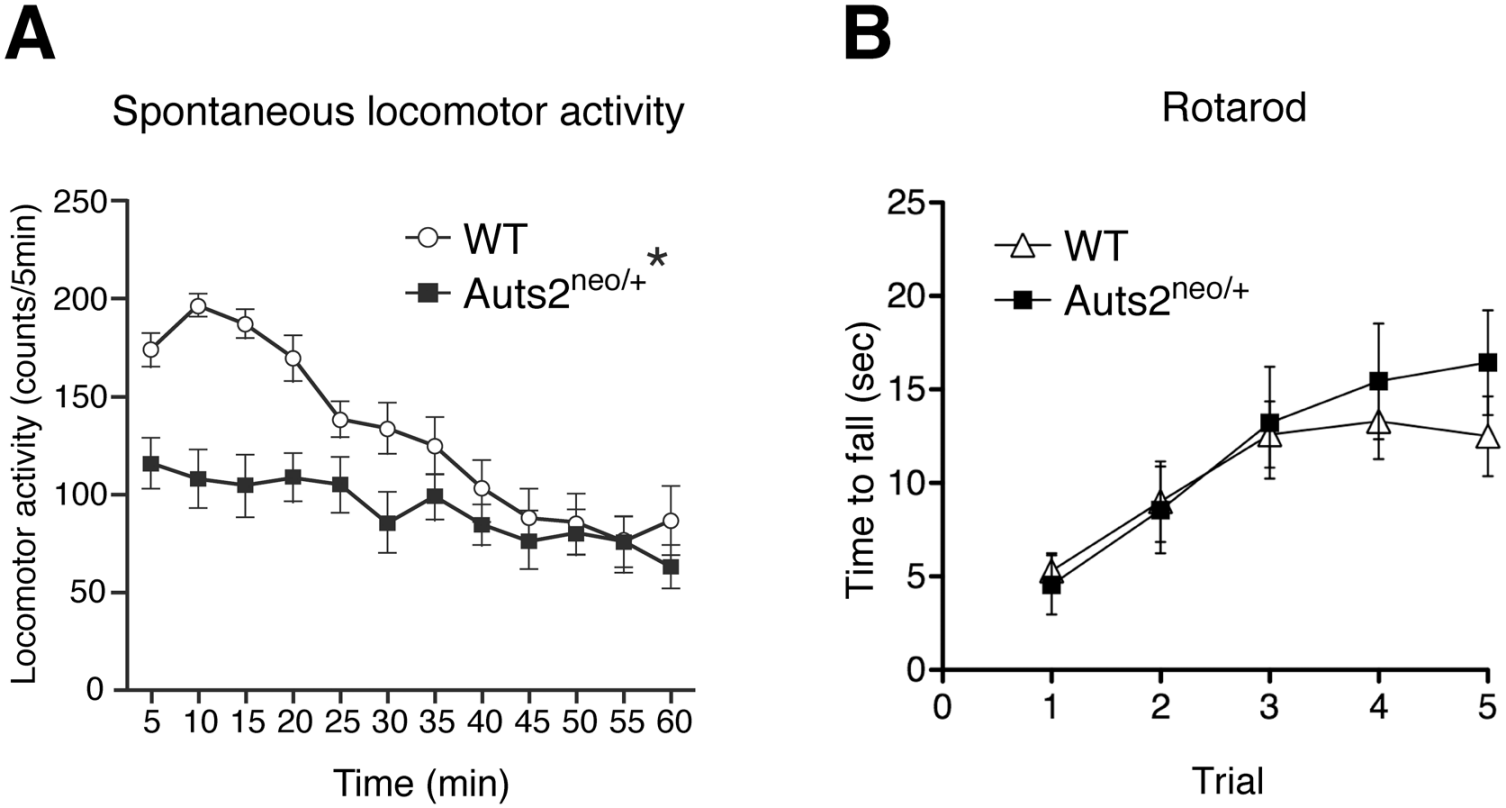
Decrease of locomotor activity in *Auts2* mutant mice during habituation. (A) Spontaneous locomotor activity for habituation to the novel environment was measured every 5 min for 60 min. Auts2-deficient mice exhibited a decrease in exploratory behavior during the first 15 min. (B) Assessment of physical motor function and coordination of Auts2 mutant mice in a rotarod test. The latency of falling from an accelerating rotarod was measured for five consecutive days. Data are presented as the mean ± SEM (A: WT, n = 17, *Auts2* ^*neo/+*^ = 14 and B: WT, n = 10, *Auts2* ^*neo/+*^ = 9). *p<0.05; two-way ANOVA with repeated measures.

When locomotor activity in a novel environment was measured for 60 min, WT mice displayed an increase in exploratory activity during the first 0–15 min, followed by a gradual reduction in activity as they became habituated to the environment (Fig 1A). Two-way ANOVA with repeated measurements revealed the significant effect of genotype [F(1,29) = 6.55, p<0.05], time [F(11,319) = 20.04, p<0.01] and interaction with genotype [F(11,319) = 6.46, p<0.01]. In comparison, *Auts2* ^*neo/+*^ mice exhibited significantly decreased exploratory behavior during the first 30 min of the test (p< 0.05, Fig 1A). However, the performance in the rotarod test was not significantly changed in *Auts2* mutants (Fig 1B: two-way repeated-measures ANOVA, genotype [F(1,17) = 0.18, p = 0.68], trial [F(4,68) = 16.74, p<0.0001] and interaction with genotype [F(4,68) = 0.92, p = 0.46]). These results suggest that reduction of exploratory behavior in the mutants is due to impairment of habituation to the novel environment, rather than defects in motor coordination.

**Fig 2:**
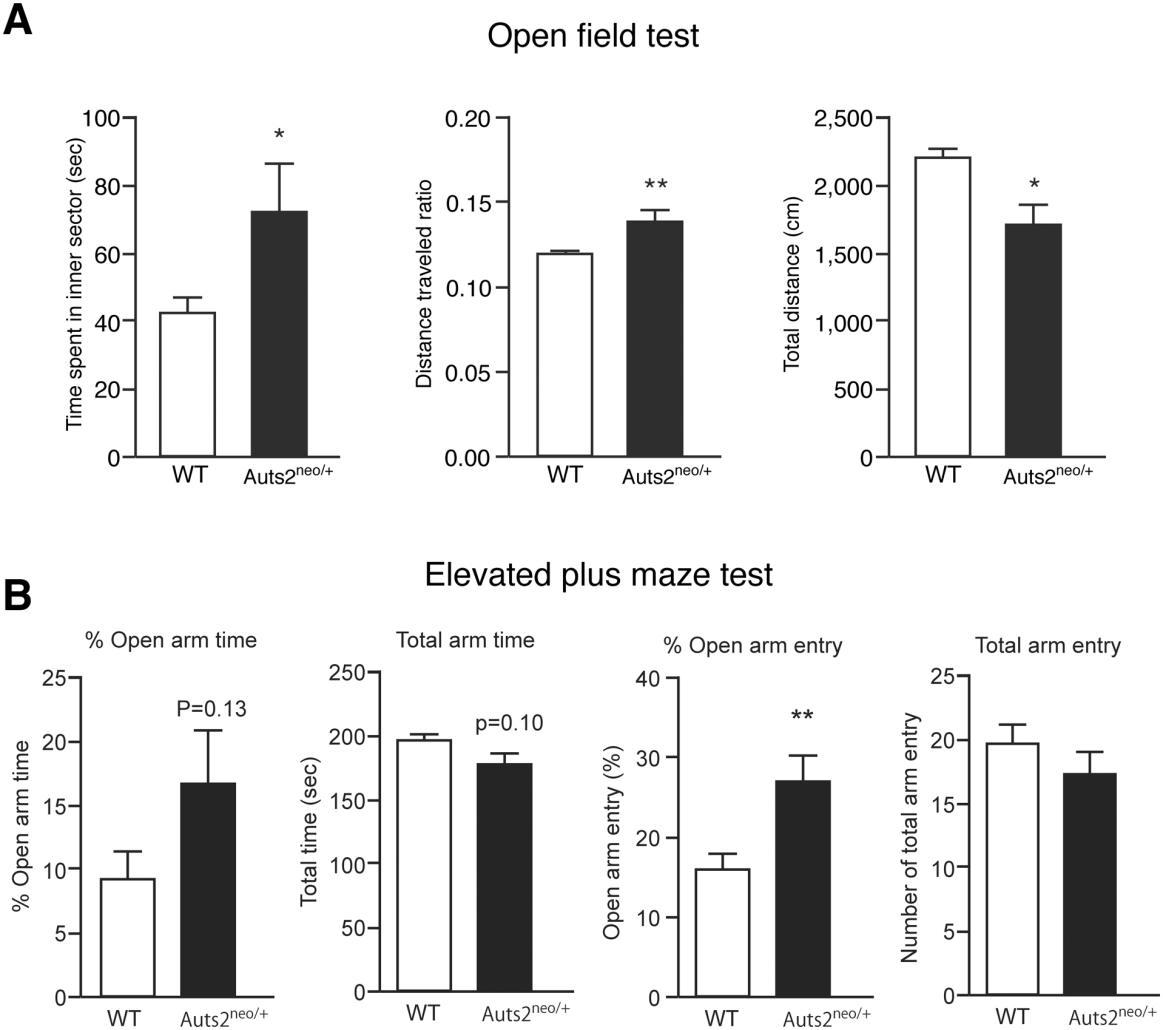
Abnormality of anxiety-like behaviors in *Auts2* mutant mice. (A) In open field tests, the time spent in the center field (left graph) and total distant traveled (right graph) were measured for 5 min to analyze mobility and exploration behavior. The ratio of distance traveled in an inner area scores as the percentage of total distance traveled (middle graph). *Auts2* ^*neo/+*^ mice spent significantly more time in the open environment than wild-type mice. (B) In elevated plus maze test, the duration of time spent in an arm (left graphs) and number of arm entries (right graphs) were measured for 5 min. The time (% Open arm time) and number of entries (% Open arm entry) in the open arms are presented by the percentage of total time (Total arm time) and number of total arm entries (Total arm entry), respectively. *Auts2* ^*neo/+*^ mice spent increased time in the unprotected open arm entry compared with wild-type mice. Data are presented as the mean ± SEM (WT, n = 17, *Auts2* ^*neo/+*^ = 14). *p<0.05, **p<0.01; Mann-Whitney U-test.

To investigate emotional behaviors in *Auts2* mutant mice, open field test was carried out, in which the conflict between the drive to explore a new environment and a natural aversion to illuminated open areas was used to examine both anxiety and motor activity. WT mice spent significantly less time in the inner sector (42.5±4.6 sec) than in the outer sector (257.6±4.6 sec) (p<0.01 by Wilcoxon matched-pairs signed-rank test, data not shown), demonstrating a natural aversion to illuminated open areas in our experimental condition (Fig 2A left). Interestingly, *Auts2* mutants spent more time in the center area compared to WT mice (Fig 2A left). The results were consistent with an increased ratio of inner vs. total distance traveled in open-field (Fig 2A middle) although a reduction of total distance traveled was observed (Fig 2A right).

The elevated plus maze test further supported the idea that *Auts2* mutants exhibit abnormal emotional behaviors. The percentage of open arm entry was markedly increased, whereas closed arm entry was significantly decreased in *Auts2* mutant mice compared with WT mice (p< 0.01 by Wilcoxon matched-pairs signed-rank test, Fig 2B, % Open arm entry). *Auts2* mutants also exhibited a marked increase, although not significant, in the percentage of open arm time as compared to WT mice (Fig 2B, % Open arm time). In contrast, motor ability seemed normal because the total time spent in either arm (Fig 2B, % total arm time) and the total frequency of entry into either arm (Fig 2B, % total arm entry) in the elevated plus maze test were not significantly different between wild type and mutant animals. These results suggest that anxiety-like behavior is decreased in the mutants.

**Fig 3:**
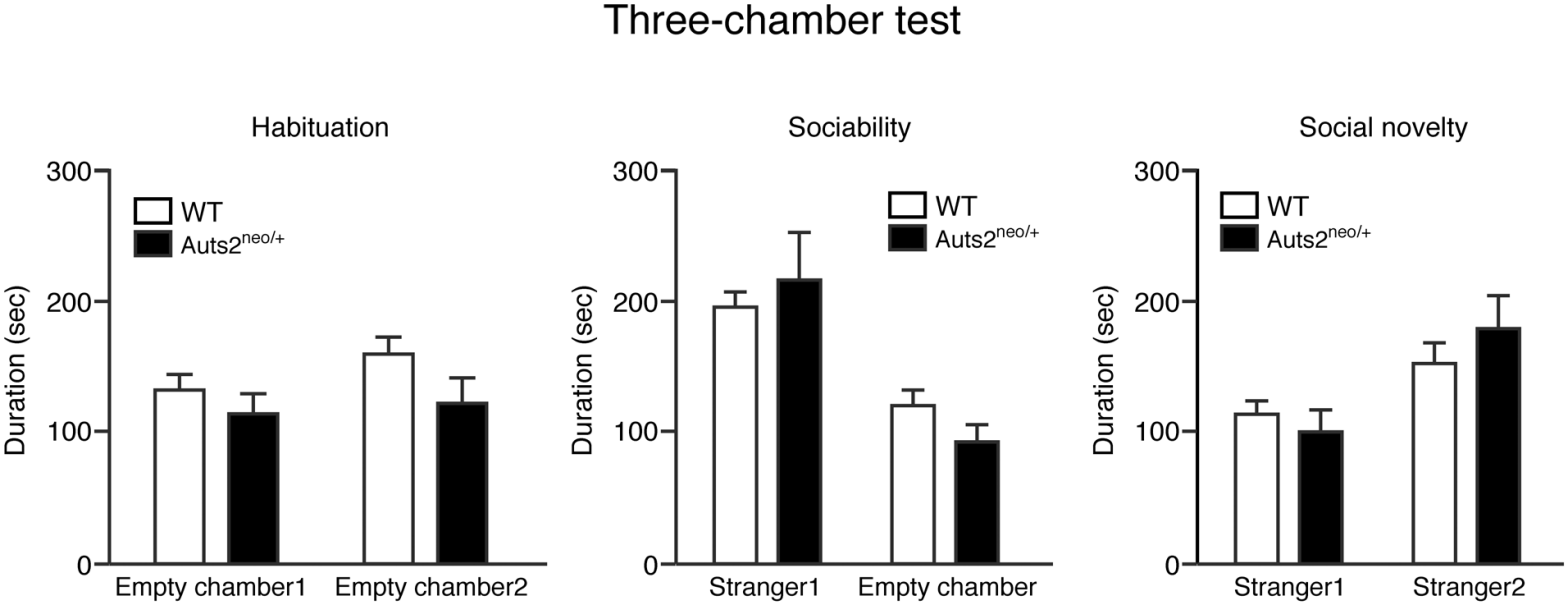
Normal social approach behaviors in *Auts2*-deficient mice. (A) Sociability for contacting an unfamiliar mouse or empty chamber (middle graphs) and social novelty for a stranger mouse versus a familiar mouse (right graphs) of WT and *Auts2* mutant mice were measured by a three-chamber test. Graphs show the time spent in the each chamber. Data are presented as the mean ± SEM (WT, n = 16, *Auts2* ^*neo/+*^ = 13), t test.

In order to investigate social approach behaviors in *Auts2* mutants relevant to human neuropsychiatric disorders, we evaluated sociability and preference for social novelty in the three-chambered social interaction test. The data from one animal in each group were excluded because they did not approach any chamber. During the habituation phase, both *Auts2* mutants and WT mice spent equal amounts of time exploring both compartments (Fig 3, left), with no biased preference for either of the two empty chambers. During the sociability phase, *Auts2* mutants and WT mice both demonstrated a preference for spending more time in the chamber containing the unfamiliar mouse (stranger 1) relative to the opposite, empty chamber [F(1,54) = 23.90, p<0.001]; there were no effects of genotype [F(1,54) = 0.03, p = 0.86], or interactions with genotype [F(1,54) = 1.39, p = 0.24] (Fig 3 middle). During the social novelty preference phase, *Auts2* mutants and WT mice also both displayed a preference for spending more time in the chamber containing the new, unfamiliar mouse (stranger 2) relative to the opposite chamber containing the previous, now familiar mouse (stranger 1) [F(1,54) = 11.22, p<0.001], and there were no effects of genotype [F(1,54) = 0.15, p = 0.70], or interactions with genotype [F(1,54) = 1.25, p = 0.27] (Fig 3 right), indicating that social approach behaviors are normal in *Auts2* mutant mice.

**Fig 4:**
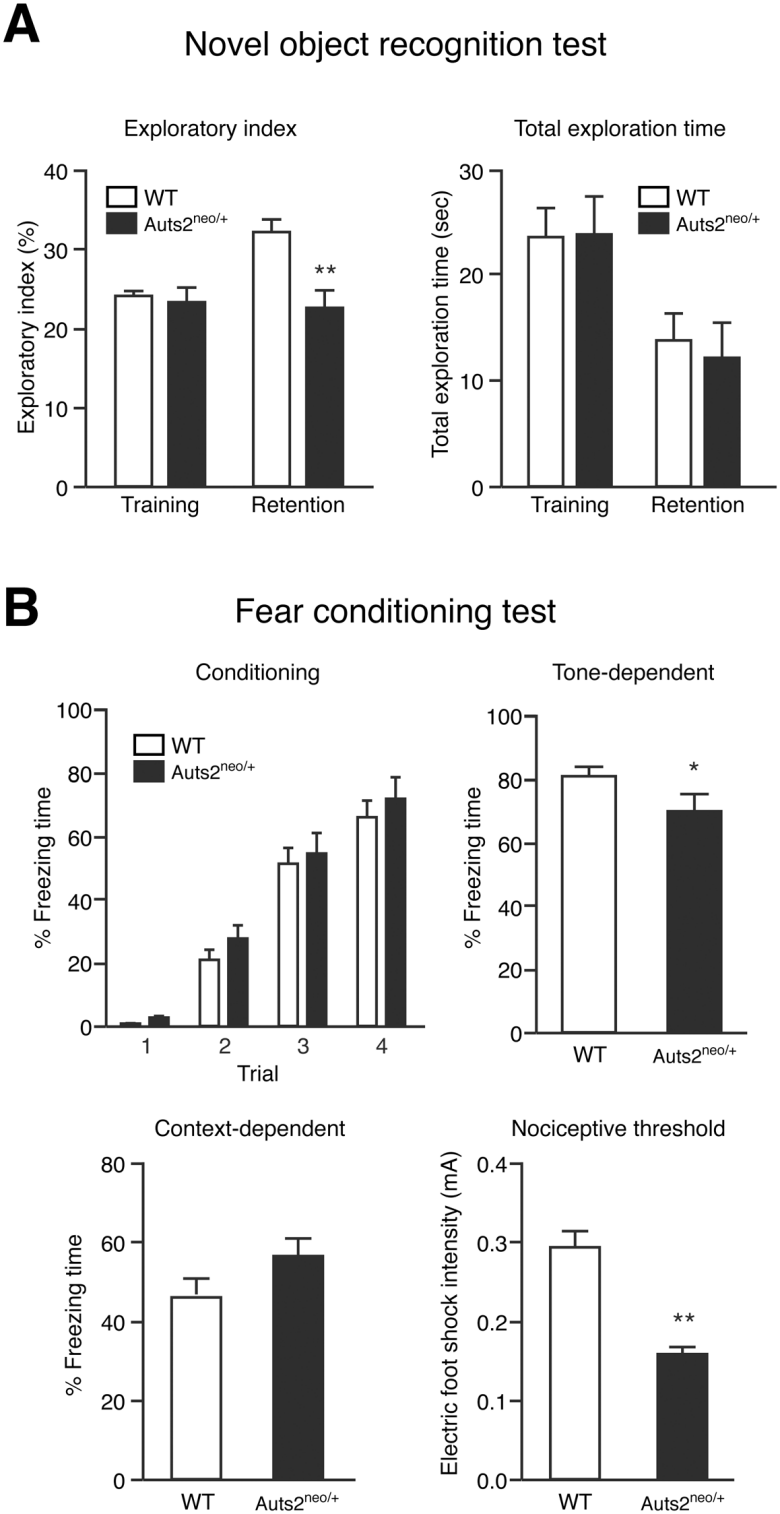
Impairment of cognitive memory functions in Auts2 mutant mice. (A) Novel object recognition test. Graphs show the exploratory preference (left graph) and total exploration time (right graph) in training and retention sessions. The retention session was carried out 24 hrs after the training session. (B) Associative memory function was measured by the cued (Tone-dependent) and contextual (Context-dependent) fear-conditioning test 24hrs after the conditioning phase (Conditioning). Note that *Auts2*-deficient mice had defective auditory fear conditioning showing a decrease of freezing response while they displayed a higher response to lower nociceptive stimuli (foot shock) compared with the wild type mice (Nociceptive threshold). Data are presented as the mean ± SEM (WT, n = 17, *Auts2* ^*neo/+*^ = 14). *p<0.05, **p<0.01; Mann-Whitney U-test.

To examine the role of Auts2 on recognition memory, a novel object recognition test was carried out. During the training session, *Auts2* mutants and WT mice spent equal amounts of time exploring the two objects (Fig 4A right), and there was no biased exploratory preference in either group (Fig 4A left), suggesting no differences in motivation and curiosity about novel objects, and motor function between *Auts2* mutants and WT mice. However, in the retention session, which was carried out 24 hrs after the training session, the level of exploratory preference to the novel object in *Auts2* mutant mice was significantly decreased compared to in WT mice (p<0.01, Fig 4A left). Total exploration time in the retention session did not differ between groups (Fig 4A right). These results suggest that *Auts2* mutant mice have impaired recognition memory.

In the fear conditioning memory test, both *Auts2* mutants and WT mice showed freezing responses during the conditioning phase, and there was no significant difference in freezing time between two groups (Fig 4B “Conditioning”). *Auts2* mutant mice showed a significant decrease in tone-dependent freezing time compared to WT mice (Fig 4B “Tone-dependent”) although there was no significant difference in the context-dependent freezing (Fig 4B “Context-dependent”). Furthermore, *Auts2* mutants showed a higher response to nociceptive stimuli (Fig 4B “Nociceptive threshold”). These findings suggest that associative memory functions are impaired in *Auts2* ^*neo/+*^ mice.

**Fig 5:**
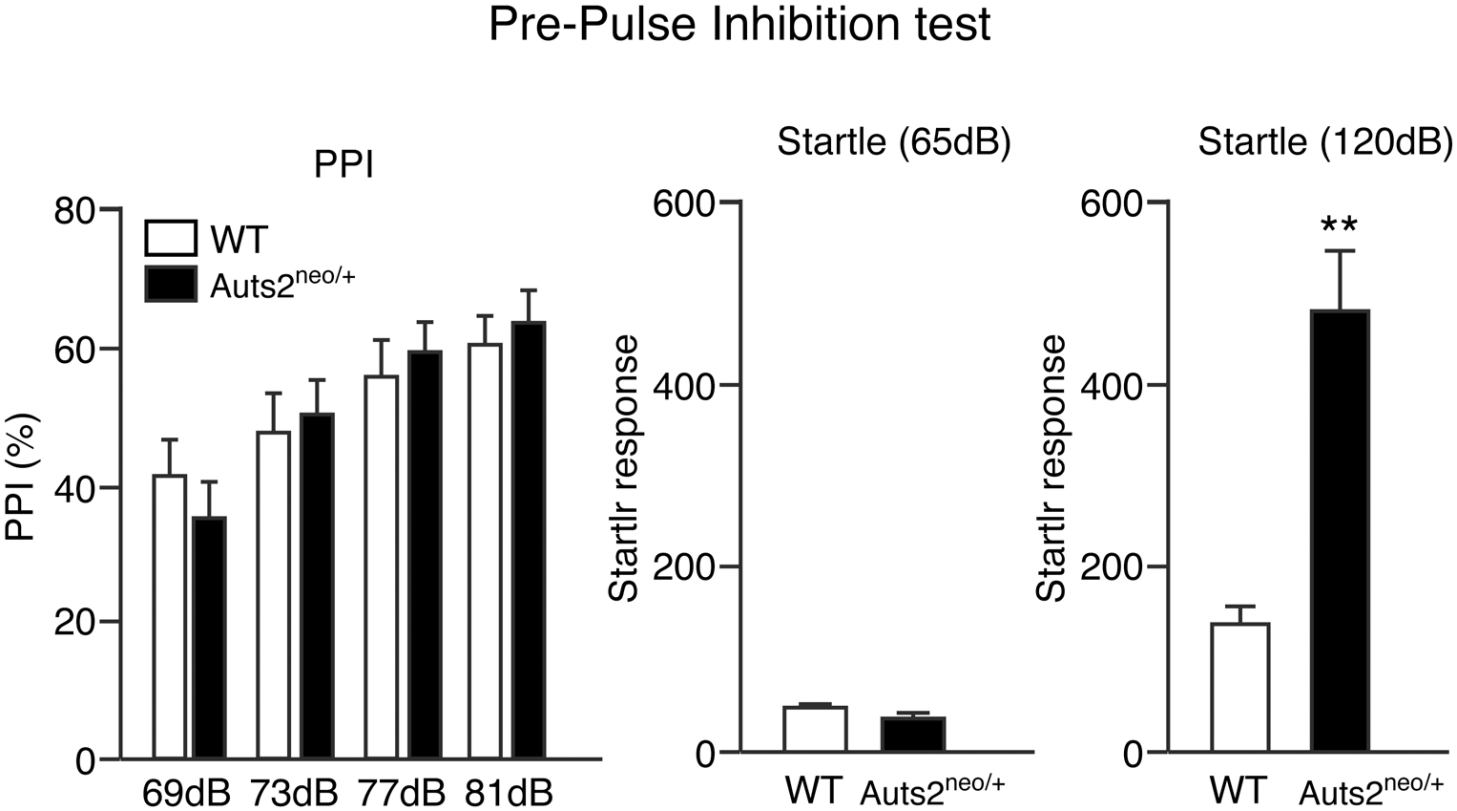
Normal behaviors of Auts2-deficient mice on PPI. PPI (%) at four different prepulse intensities (69, 73, 77 and 81 dB) in PPI test (left graph) and acoustic startle response to two different pulses (middle and right graphs) as measured in trials without a prepulse. There were no significant differences between wild type and *Auts2* mutant mice in the characterization of PPI whereas *Auts2* mutant mice exhibited a higher acoustic startle response at 120 dB pulse compared to WT mice. Data are presented as the mean ± SEM (WT, n = 17, *Auts2* ^*neo/+*^ = 13). **p<0.0001; two-way ANOVA with repeated measures in PPI test and Mann-Whitney U-test in startle response.

We further examined the acoustic startle response and the percentage of prepulse inhibition (PPI) as indicators of sensorimotor processing. The data obtained from one *Auts2* mutant was excluded because of no response to startle stimulus. Repeated measures ANOVA of the data (69–81 dB) revealed a significant effect of prepulse intensity [F(3,84) = 17.75, p<0.01]. There were no effects of genotype [F(1,28) = 0.03, p = 0.87], or interaction of genotype [F(3,84) = 0.82, p = 0.49] (Fig 5 left). However, *Auts2* mutants displayed a potentiation of acoustic startle response at 120 dB compared to WT mice (p<0.01, Fig 5 right).

Together, these results suggest that AUTS2 plays an important role for high-order brain functions such as anxiety-related emotional behaviors as well as in certain types of memory formation.

## Discussion

Many studies have previously demonstrated that *AUTS2* was implicated as a promising candidate for multiple neurocognitive defects as well as developmental delay and epilepsy [2, 4]. In those reports, although some cases were reported to have disrupted *AUTS2* coding regions, many other cases were exclusively structural variants in which *AUTS2* was disrupted by genomic rearrangements including balanced translocation, inversions or deletions of *AUTS2* non-coding regions, implying the possibility that other genes could be involved in the occurrence of the disorders. Recently, however, Beunders et al. have identified a significant number of individuals with ASD and/or ID having microdeletions in which at least one exon of *AUTS2* was included [7]. Consistent with human cases, we found in this study that heterozygotic disruption of *Auts2* alone sufficiently induced several behavioral abnormalities including decrease of exploratory behaviors in a new circumstance as well as the decrease of anxiety against aversive situations for mice. Furthermore, *Auts2*-deficient mice displayed learning defects related to recognition and associative memory, suggesting that AUTS2 contributes to the acquisition of neurocognitive brain function. Interestingly, *Auts2*-deficient mice exhibited higher responses against nociceptive (Fig 4B “Nociceptive threshold”) and acoustic stimuli (Fig 5 right) while social behavior was normal in three-chambered assay (Fig 3 middle and right). In human patients with autism, hypersensitivity against auditory and tactile stimuli is commonly reported in addition to the triad of impairments [24]. Together, these findings indicate a possibility that these heterozygous animals mimic in part the pathology of some patients with *AUTS2* mutations.

Previous studies have demonstrated that mutations in the genes involved in the regulation of cytoskeleton organization were associated with mental retardation, epilepsy and learning disorders [25, 26]. For example, disruption of microtubule organizer genes such as Lis1, Nde1, DCX and FGF13 in human and mouse models result in defects of neuronal migration or aberrant neurite formation and also exhibit impaired learning and memory [27–34]. It has been reported that individuals with heterozygotic disruptions of the human *AUTS2* occasionally present with brain malformations including microcephaly and corpus callosum hypoplasia [3, 35]. In our recent study, although *Auts2*-deficient mice do not show significant gross morphological and histological abnormalities in brain structure, several developmental processes including the cortical neuronal migration and subsequent axon development were impaired in both homozygotic and heterozygotic *Auts2* mutant mice d[16]. The defects in learning and memory of *Auts2* mutant mice could be caused by the developmental abnormalities in the microstructure within the brain, which may hamper the establishment of proper neural circuits in the mature brain. Additionally, AUTS2 may also be involved in the postnatal neurodevelopmental processes such as synaptogenesis, synaptic neurotransmission and neural circuit formation because AUTS2 is continuously expressed in the mature mice brain. Future studies of Auts2 mutant mice will allow us to better understand the function of AUTS2 in these processes.
